# Effects of frequently used psychiatric drugs on subjective dry mouth, salivary secretion and DMFT in outpatients

**DOI:** 10.1186/s12903-026-07958-8

**Published:** 2026-02-28

**Authors:** Alexandra Kovács, Katalin Károlyházy, Judit Tolna, Laura Gótai, Tamás Demeter, Beáta Kerémi, János Vág, Krisztina Márton

**Affiliations:** 1 Department of Restorative Dentistry and Endodontics, Faculty of Dentistry, Semmelweis University, Budapest, Hungary; 2 Department of Prosthodontics, Faculty of Dentistry, Semmelweis University, Budapest, Hungary; 3 Department of Psychiatry and Psychotherapy, Faculty of Medicine, Semmelweis University, Budapest, Hungary; 4 Rosental Dental and Implantology Clinic, Budapest, Hungary; 5 Department of Preclinical Dentistry, Faculty of Dentistry, Semmelweis University , Budapest, Hungary

**Keywords:** Xerostomia, Mouth dryness, Drug therapy, Psychiatric diseases, Mental disorders, Psychiatric medications

## Abstract

**Background:**

Psychiatric disorders are commonly treated with medications that can alter salivary gland function and lead to xerostomia. Reduced saliva flow and increased oral dryness can significantly impact oral health and quality of life. The aim of this study was to evaluate both subjective and objective indicators of salivary gland function among psychiatric outpatients in Hungary and to explore the potential xerogenic effects of the most frequently prescribed psychiatric drugs.

**Method:**

A total of 188 psychiatric outpatients (70 men, 118 women) and 173 age- and sex-matched healthy controls (80 men, 93 women) were examined. Subjective sicca symptoms were assessed using a 16-question xerostomia questionnaire. Objective measurements included the determination of unstimulated whole salivary secretion rate and minor salivary gland secretion rates (palatal and labial), and dental caries experience was evaluated using the DMFT index. Descriptive statistics and multiple linear regression analyses were performed to assess the effects of demographic and medication variables on salivary parameters and DMFT.

**Results:**

Psychiatric patients exhibited lower unstimulated whole salivary secretion rate (females) and labial minor salivary gland secretion rates, alongside higher xerostomia frequency and DMFT values, compared with controls. The most commonly prescribed drugs were benzodiazepines (80%) and atypical antipsychotics (67%), with frequent concomitant use of multiple psychiatric medications. Regression analyses identified age and psychiatric group as predictors of reduced salivary secretion and increased DMFT scores. Typical antipsychotic use showed a weak positive association with unstimulated whole salivary secretion rate, while benzodiazepine and lithium use correlated with higher palatal minor salivary gland secretion. Age strongly predicted increased DMFT.

**Conclusions:**

Psychiatric patients demonstrated impaired salivary gland function and poorer dental health compared with healthy individuals. While the associations between specific drug classes and salivary parameters were mostly weak, long-term psychopharmacological treatment and drug combinations appear to contribute to the high prevalence of xerostomia in this population. Awareness of these effects is essential for implementing preventive oral care strategies in psychiatric patients. A potential limitation of the study is that unstimulated saliva collection in control participants was performed during routine dental visits, where situational stress may have transiently influenced flow rates.

**Trial registration:**

This study was retrospectively registered at ClinicalTrials.gov (NCT06920472; public release: 05/19/2025).

## Background

Patients with mental disorders are known to have poorer oral health than the general population [1]. This multifactorial vulnerability arises from dental phobia, self-neglect, cognitive impairment, the oral adverse effects of psychiatric medications, and social challenges such as unemployment, isolation, and poverty [2]. Unhealthy dietary habits, increased sugar intake, and the consumption of alcohol, tobacco, and recreational drugs further contribute to unsatisfactory oral health [3]. At the same time, psychiatric disorders are a major and growing public health concern, with prevalence in the European Union rising from 27.4% in 2005 to 38.2% in 2010 [4], and recent data confirming high rates of depressive symptoms across the lifespan [5, 6]. As pharmacological treatment has expanded [7], chronic use of psychiatric medications has become increasingly common. Although essential for symptom control, a lot of psychotropic drugs cause oral side effects—most notably salivary hypofunction and xerostomia [8, 9]— which increase the risk of dental diseases and complicate dental management. However, in the context of drug-induced xerostomia, the relationship between objectively measured salivary flow rates and subjective perceptions of oral dryness or drooling remains unclear [9]. These factors underscore the urgent need to clarify how specific psychiatric drug classes affect oral dryness, salivary secretion, and caries risk, to implement targeted preventive strategies and improve oral care for this vulnerable group of patients.

Psychiatric drugs can cause alterations of the saliva flow rate via the vegetative nervous system, activating M1- M3-receptors, α1- and β1-adrenergic-and some peptidergic receptors, which can result in hyper- or hyposalivation [10, 11].

Hyposalivation can lead to severe oral consequences, including increased caries prevalence, impaired speech and swallowing, and gastrointestinal complaints [12–14]. Psychiatric medications are widely recognized contributors to xerostomia; however, their effects on objectively measured salivary secretion remain inconsistent across drug classes and studies [9, 15–20]. While earlier antidepressants and antipsychotics have been strongly associated with reduced salivary flow, findings related to newer-generation antidepressants and atypical antipsychotics - characterized by broader receptor profiles and reduced extrapyramidal side effects compared to typical antipsychotics - are more heterogeneous [21–30]. Importantly, patients with psychiatric disorders consistently demonstrate poorer oral health outcomes, including higher DMFT scores, compared with healthy populations across different countries [1, 31, 32]. Furthermore, meaningful associations have been reported between depression, anxiety, elevated psychological stress, and tooth loss, as well as between depression and increased numbers of decayed teeth in adult populations [33–35]. These observations highlight the need for studies combining subjective symptoms with objective salivary measurements and oral hygiene in psychiatric populations.

The aim of the present study was to determine the effects of the most frequently prescribed psychiatric drugs on subjective oral dryness, whole- and minor saliva secretion, and DMFT in psychiatric outpatients.

## Materials and methods

The present study was retrospectively registered at ClinicalTrials.gov (NCT06920472). The registration was completed after data collection but before manuscript submission, following the ethical and reporting standards for human clinical research. The present study was affirmed by the Medical Research Council, Scientific and Research Committee of Hungary (ETT-TUKEB) No: 55/2013, and was conducted in accordance with according to the Helsinki Declaration of Human Rights. In the psychiatric outpatient group, the capacity of making decisions was evaluated by a supervising mental health professional, and only those deemed capable of ethically and medically providing informed consent were included in the study. All healthy control participants provided written informed consent their participation in the study.

### Patients

One hundred eighty-eight psychiatric patients (70 men and 118 women) have been investigated at the Outpatient Clinic, Department of Psychiatry and Psychotherapy, Semmelweis University, Budapest, Hungary. Psychiatric outpatients were recruited consecutively over a one-year period during routine clinical care, and eligibility was determined by the treating psychiatrist based on predefined inclusion and exclusion criteria, including the assessment of decision-making capacity. Patients deemed unsuitable for participation of clinical or ethical reasons were not enrolled and were not registered separately. In the psychiatric group, inclusion criteria comprised ongoing treatment with at least one of the following psychiatric drug groups: serotonin antagonists and reuptake inhibitors (SARI), selective serotonin reuptake inhibitors (SSRI), serotonin–norepinephrine reuptake inhibitors (SNRI), noradrenergic and specific serotonergic antidepressants (NASSA), atypical antipsychotics (AA), typical antipsychotics (TA), valproate (VPA), benzodiazepines (BDZ), lithium (LI), or tricyclic antidepressants (TCA). Psychiatric diagnoses were classified according to the DSM-5 diagnostic criteria. The diagnostic categories listed above represent the most frequent diagnoses within the psychiatric cohort. Not all participants consented to the recording of detailed diagnostic information as part of the study. Among the psychiatric patients, depressive disorders (53% in women, 30% in men), **s**chizophrenia spectrum disorders (12% in women, 30% in men), bipolar and related disorders (8% in women, 14% in men), and anxiety disorders (24% in women, 8% in men) were the most frequent diagnoses.

As a comparison group, 173 age- and sex-matched healthy control participants (80 men and 93 women) were recruited randomly from individuals attending the Educational Centre, Faculty of Dentistry, Semmelweis University, Budapest, Hungary, including patients registering for routine dental care. In the control group, exclusion criteria included any self-reported psychiatric disorder, other systemic diseases, or the use of regular medication. Health status and eligibility were assessed on the day of saliva collection by structured self-report questionnaire and interview.

### Most commonly prescribed psychiatric medications

Xerogenic side effects of the following psychiatric drugs were examined: SARI, SSRI, SNRI, NASSA, SNRI+NASSA, AA, TA, VPA, BDZ, LI and TCA. See Table 1 for the effectivity fields, acting mechanisms, and possible side effects of the different drugs and frequencies of participants, the gender ratio within the drugs. 

**Table 1:** Overview of Psychiatric Medication Groups Used by Psychiatric Patients

Drug groups	Number of participants (*n* = 188) [ratio of men/women]	Active agent	Treatment indication	Mechanism of action	Known medication side effects
Serotonin Antagonists and Reuptake Inhibitors (SARI)	17 [10/7]	Trazodon, Etoperidone, Lorpiprazole, Mepiprazole, Nefazodone	Depression	Inhibits the reuptake of 5-HT and activation of 5-HT_2_- and α_1_-receptors	Sedation, vertigo, orthostatic hypotension, nausea
Selective Serotonin Reuptake Inhibitors (SSRI)	40 [8/32]	Fluoxetine, Fluvoxamine, Paroxetine, Sertralin, Citalopram, Escitalopram	Depression, anxiety disorders	Inhibits the reuptake of 5-HT (selective for the serotonin transporter)	Anxiety, excitement, headache, nausea, vomitus, diarrhoea, sexual disfunction
Serotonin and Norepinephrine Reuptake Inhibitors (SNRI)	30 [11/19]	Venlafaxin, Duloxetine	Depression, mood affective disorders, anxiety disorder, neuropathic pain	Inhibits presynaptic reuptake of NA and 5-HT	Similar to SSRI; in big dosage: high blood pressure
Noradrenergic and Specific Serotonergic Antidepressants (NASSA)	21 [7/14]	Mirtazapine, Mianserin, Aptazapine, Esmirtazapine	Depression	Inhibits presynaptic α_2_-autoreceptor, H_1_-muscarinergic- and α_1_-receptor	Gain in weight, sedation
Atypical Antipsychotics (AA)	124 [45/79]	Amisulpride, Clozapine, Olanzapine, Paliperidone, Risperidone, Quetiapine	Schizophrenia	Integrate with the 5-HT- α-, β- and D-receptors	Extrapyramidal symptoms (acute dystonia, akathisia, movement disorders, perioral tremor)
Typical Antipsychotics (TA)	19 [8/11]	Fluphenazine, Haloperidol, Chlorpromazine	Psychosis, schizophrenia	Integrate with the 5-HT- α-, β- and D- receptors	Extrapyramidal symptoms
Valproate (VPA)	19 [10/9]	-	Bipolar affective disorder, epilepsy	Inhibits T-type calcium - and voltage-gated natrium channels	Nausea, drowsiness, dizziness, vomiting, weakness, bleeding, low blood platelets
Benzodiazepine (BDZ)	150 [60/90]	Alparzolam, Clonazepam, Lorazepam, Midazolam, Nitrazepam	Epilepsy, panic- and anxiety disorder	Increase GABAerg inhibition (allosteric modulation of GABA-A receptor)	Sedation, ataxia, dependence
Lithium (LI)	26 [9/17]	-	Bipolar depression, manic depressive disorder	Not clearly known	Nausea, vomiting, abdominal pain, drowsiness, gaining weight
Tricyclic Antidepressants (TCA)	11 [6/5]	Imipramine, Amitriptyline, Clomipramine, Trimipramine, Dibenzepine	Depression	Inhibits the reuptake of NA and 5-HT	**Xerostomia**, obstipation, visual disorder, tachycardia

The table summarizes the main psychotropic drug classes prescribed to psychiatric outpatients, including the number of participants by sex, the most frequently used active agents within each group, their primary therapeutic indications, principal pharmacological mechanisms of action, and commonly reported adverse effects

*Abbreviations*: *5-HT*serotonin, *5-HT*_*2*_5-hydroxytryptamine 2 receptor, *α*_*1*_ alfa_1_adrenergic receptor, *α*: alfa adrenergic receptor,β: beta adrenergic receptor, *H*_*1*_ histamine type 1 receptor, *D*: Dopamine receptor, *NA*: Noradrenaline, *GABA*: Gamma-Aminobutyric Acid

### Assessment of the orofacial and extra-oral sicca symptoms

The prevalence of xerostomia and other associated subjective sicca symptoms was assessed using a questionnaire developed within our xerostomia research group and previously applied in Hungarian clinical populations. (16 questions total, in Table 2) [36]. Responses were recorded as yes or no, and if present, symptom severity was graded as mild, moderate, or severe.

**Table 2:** Xerostomia and other associated subjective sicca symptoms questionnaire

Questions
1. Does your mouth often feel dry?
2. Do you find it difficult to swallow?
3. Does your tongue often tingle or have a burning sensation?
4. Is your speech significantly affected, if you talk for a long period?
5. Do you have problems when tasting?
6. Do you feel not having enough saliva in your mouth?
7. Does your saliva often feel thick?
8. Do your teeth easily decay?
9. Does your nose dry out sometimes?
10. Do your eyes often feel dry?
11. Do your eyes often itch, burn or tingle or you have the feeling of having something inside?
12. Are you sensitive to light?
13. Does your skin often feel dry?
14. Do you suffer from vaginal dryness?
15. Does your vagina sometimes itch, burn? Do you suffer from recurrent vaginal fungal infection?
16. Do you often feel tired?

The table presents the items of the questionnaire used to assess subjective oral and extra-oral sicca symptoms among study participants. Participants were instructed to answer each question with “Yes” or “No”; if “Yes”, symptom severity was further graded as mild, moderate, or severe. The questionnaire was administered to all participants prior to the clinical examinations

### Clinical examinations

Clinical oral examinations were performed by a trained dental examiner for all participants during the study period. Dental caries experience was assessed and recorded using the DMFT index at the time of the clinical examination.

### Exocrine function tests

Unstimulated whole salivary secretion rate (UWS) and minor salivary gland (MSG) secretions were measured under standardized conditions. All saliva collections were performed during daytime hours (morning and early afternoon). Participants were instructed to refrain from eating, drinking, having a chewing gum and smoking for at least 1 h prior to saliva collection. UWS was determined using the “spitting” method [37, 38]. Whole saliva was collected for 5 min into a previously weighed empty cup and then measured again after collecting the saliva. Obtained data were expressed in ml/min. Values 0.1 ml/min or less were considered as hyposalivation.

Palatal and labial minor salivary gland (MSG) flow rates were measured using a Periotron 8010^®^ device (Oraflow Inc., Amityville, USA). Prior to saliva collection, the Periotron device was calibrated and zeroed using a dry filter paper disc. Minor salivary gland secretion was measured using standardized filter paper discs (8 mm diameter; surface area 0.5 cm²). Filter paper discs were placed on the hard palate at the level of the upper first molar, approximately 15 mm from the gingival margin and on the inner surface of the lower lip near the midline. Before placement, the sampling areas were dried and isolated with cotton rolls. The discs were kept in position for 30 s [39, 40]. After collection, the wetted filter paper discs were immediately reinserted into the Periotron device, and the digital readout corresponding to the amount of absorbed saliva was recorded. The Periotron measures changes in capacitance through the wetted disc, which correlate with the volume of absorbed saliva [41]. The Periotron readings were then transformed into salivary flow rates expressed as µl/cm²/min using the device-specific calibration curve, based on prior calibration of the Periotron with known quantities of distilled water, and normalized for collection time [39].

### Statistical analysis

Descriptive statistics (median, IQR, and range) were calculated for continuous variables, and frequencies and percentages were used for categorical data. The distribution of outcome variables was evaluated using histograms and normal probability plots. Regression assumptions were assessed using residual diagnostics rather than raw variable distributions. Multiple linear regression analyses were carried out to examine the effects of predictors such as sex, age, and various medication uses on UWS-, MSG secretion, DMFT index. Their standardized residuals showed approximately non-normal distributions for all outcome variables, and independence of residuals was confirmed using the Durbin–Watson statistic (range: 1.53–1.65). The strength and direction of associations were evaluated using partial correlation and corresponding p-values, with statistical significance set at *p* < 0.05. Correlations were interpreted as weak (partial-*r* < 0.3), moderate (0.3 ≤ partial-*r* < 0.6), or strong (partial-*r* ≥ 0.6), following conventional thresholds. No formal correction for multiple testing was applied, as the regression analyses were hypothesis-driven with predictors selected a priori and entered simultaneously into multivariable models; inference was based on effect estimates and confidence intervals rather than isolated p-values. Given the exploratory nature of medication-related predictors, findings should be interpreted with consideration of effect sizes and confidence intervals rather than p-values alone.

## Results

The median age of the female psychiatric group was 50.5 (IQR:35.25-59; range: 18–83) while the control female group had a median age of 46** (**IQR: 24–61; range: 20–78) (Table 5). The median age of the male psychiatric group was 42.5(IQR: 33-54.75; range: 19–76), the control male group had a median age of 38.5(IQR: 24-62.25; range: 19–75) respectively (Table 5).

The distribution of psychiatric medication use among psychiatric patients can be seen in Table 3. The most frequently used drug categories were benzodiazepines (*n* = 150, 90 in female and 60 in male) and atypical antipsychotics (*n* = 124, 79 in female and 45 in male) in both sexes. The majority of psychiatric patients were prescribed combinations of two or more psychiatric drugs, indicating a high prevalence of concomitant use of multiple psychiatric medications among patients under active psychiatric treatment.

**Table 3:** Medication use frequency among test group participants

Drug	Female (*n*/%)	Male (*n*/%)	Total (*n*/%)
SARI	7 (4%)	10 (5%)	17 (9%)
SSRI	32 (18%)	8 (4%)	40 (22%)
SNRI	19 (10%)	11 (6%)	40 (16%)
NaSSA	14 (7%)	7 (4%)	21(11%)
SNRI+NASSA	4 (2%)	3 (2%)	7 (4%)
AA	79 (42%)	45 (25%)	124 (67%)
TA	11 (6%)	8 (4%)	19 (10%)
VPA	9 (5%)	10 (5%)	19 (10%)
BDZ	90 (48%)	60 (32%)	150 (80%)
LI	17 (9%)	9 (5%)	28 (14%)
TCA	5 (3%)	6 (3%)	11 (6%)

The table presents the frequency of prescribed psychiatric medication classes in the psychiatric (test) group, stratified by sex. Values are expressed as the number of participants (n) and percentage (%). Individual participants may be represented in more than one medication category due to concomitant use of multiple psychiatric medications.

Abbreviations: *n* number of participants, *SARI* Serotonin Antagonists and Reuptake Inhibitors, *SSRI* Selective Serotonin Reuptake Inhibitors, *SNRI* Serotonin–Norepinephrine Reuptake Inhibitors, *NASSA* Noradrenergic and Specific Serotonergic Antidepressants, *AA* Atypical Antipsychotics, *TA* Typical Antipsychotics, *VPA* Valproate, *BDZ* Benzodiazepine, *LI* Lithium, *TCA* Tricyclic Antidepressants

All participants in both the psychiatric (test) and control groups completed the Xerostomia Questionnaire, allowing the comprehensive evaluation of subjective sicca symptoms. According to the xerostomia questionnaire, the most frequently reported sicca symptoms were oral dryness and perceived caries susceptibility, while fatigue and dry skin were also commonly mentioned complaints. Table 4 summarizes the responses to Question 1 and Question 8, as these items directly correspond to the two composite indices used in later analyses: Question 1 informs the Xerostomia Frequency (XF) score, while Question 8 reflects the Perceived Caries Susceptibility (PCS) score summarized in Table 7. A high proportion of psychiatric patients reported subjective oral dryness in both sexes. Among females, 60.2% of psychiatric patients reported any grade of xerostomia (controls only 33.3%). Respectively, 50% of male psychiatric patients indicated some degree of oral dryness (31.2% of male controls). Perceived caries susceptibility was also more frequent among psychiatric participants: approximately 78% of female and 80% of male patients reported that their teeth decay easily (controls: 70% in females, 60% in males). These findings highlight a greater prevalence and severity of xerostomia and self-reported dental health in the psychiatric group compared with the control population.

**Table 4:** Subjective Reports of Dry Mouth and Perceived Caries Susceptibility (Xerostomia Questionnaire, Question 1 and 8)

Xerostomia questionnaire			Female				Male			
			Test (*n* = 118)		Control (*n* = 93)		Test (*n* = 70)		Control (*n* = 80)	
1. Does your mouth often feel dry?	No		47 (39.8%)		62 (66.7%)		35 (50%)		55 (68.8%)	
	Yes	*mildly*	22 (18.6%)	71 (60.2%)	15 (16.1%)	31 (33.3%)	10 (14.3%)	35 (50%)	16 (20%)	25 (31.2%)
		*moderately*	33 (28%)		12 (12.9%)		15 (21.4%)		7 (8.7%)	
		*severely*	16 (13.6%)		4 (4.3%)		10 (14.3%)		2 (2.5%)	
8. Do your teeth easily decay?	No		26 (22%)		20 (21.5%)		14 (20%)		24 (30%)	
	Yes	mildly	21 (17.8%)	92 (78%)	15 (16.1%)	73 (78.5%)	14 (20%)	56 (80%)	15 (18.7%)	56 (70%)
		moderately	36 (30.5%)		36 (38.7%)		25 (35.7%)		24 (30%)	
		severely	35 (29.7%)		22 (23.7%)		17 (24.3%)		17 (21.3%)	

The table presents responses to Question 1 (“Does your mouth often feel dry?”) and Question 8 (“Do your teeth easily decay?”) of the xerostomia questionnaire, stratified by sex and study group. Only these two items are shown because they directly correspond to the composite scores used in subsequent analyses: Question 1 contributes to the Xerostomia Frequency (XF) score, and Question 8 reflects Perceived Caries Susceptibility (PCS). Participants subjectively reported the presence or absence of symptoms; positive responses were further categorized according to perceived severity (mild, moderate, or severe). Values are expressed as number of participants (n) and percentage (%). *Abbreviations*: *n* number of participants

In the female control group, one participant declined to provide UWS and MSG palatal samples and did not permit dental examination for DMFT assessment. Similarly, in the female psychiatric group, one participant refused UWS and both MSG samples, and did not allow DMFT data collection. In the male test group, two participants were unwilling to provide UWS and MSG samples. These missing data explain the small variation in participant numbers (n) presented in Table 5.

**Table 5:** Descriptive statistics of salivary parameters and DMFT

Sex	Parameter	Group	*n*	Median	IQR	Range
Female	Age	Control	93	46	24–61	20–78
		Test	118	50.5	35.25-59	18–83
	UWS (ml/min)	Control	92	0.12	0.07–0.22	0–0.9
		Test	117	0.08	0.03–0.17	0–1.52
	MSG labial(µl/cm^2^/min)	Control	92	2.2	0.6–3.25	0.15–11.7
		Test	117	1.13	0.53–2.3	0.23–10.8
	MSG palatal(µl/cm^2^/min)	Control	92	1.09	0.45–1.6	0.1–15.2
		Test	117	1.1	0.46–2.4	0.2–17.5
	DMFT	Control	92	14	8.75-21	2–28
		Test	117	17	12–25	2–28
Male	Age	Control	80	38.5	24-62.25	19–75
		Test	70	42.5	33-54.75	19–76
	UWS (ml/min)	Control	80	0.13	0.07–0.29	0.02–1.93
		Test	68	0.15	0.08–0.29	0–0.93
	MSG labial(µl/cm^2^/min)	Control	80	1.86	0.64–4.11	0-17.7
		Test	68	1.3	0.78–2.7	0.18–9.7
	MSG palatal(µl/cm^2^/min)	Control	80	1.12	0.56–1.76	0-8.8
		Test	68	1.56	0.66–2.59	0.2–9.7
	DMFT	Control	80	14	7–19	1–28
		Test	68	16.5	10–21	1–28

The table summarizes descriptive statistics (mean, standard deviation, and range) for age, unstimulated whole salivary secretion rate (UWS), minor salivary gland (MSG) secretion rates (labial and palatal), and the DMFT index in psychiatric (test) and control participants, stratified by sex. Differences in sample size (*n*) across parameters reflect occasional missing measurements due to participant refusal. All values are presented as mean ± standard deviation, with the observed range indicated

*Abbreviations*: *n* number of participants, *SD* standard deviation, *DMFT* Decayed, Missing and Filled Teeth index

Psychiatric patients exhibited generally lower UWS (females) and MSG secretion rates compared to healthy controls, while mean DMFT values were higher in both genders.

Among females, the median UWS was 0.08 ml/min (IQR: 0.03–0.17 ml/min) in the test group and 0.12 ml/min (IQR: 0.07–0.22 ml/min) in controls, and among males 0.15 ml/min (IQR: 0.08–0.29 ml/min) and 0.13 ml/min (IQR: 0.07–0.29ml/min), respectively. When applying the conventional cut-off for unstimulated whole saliva, hyposalivation was observed in 64 female and 26 male psychiatric patients, compared with 36 female and 33 male participants in the control group. MSG secretion rates showed a similar trend, with lower labial values in psychiatric participants. However, the test group exhibited higher palatal MSG secretion rates compared to controls. This finding may be influenced by site-specific measurement variability at the palatal sampling location, as well as by potential differences in secretory behaviour among minor salivary gland types [42]. The median DMFT index was higher in the psychiatric group for both sexes (median and IQR; female: 17 and 12–25; male: 16.5 and 10–21) compared to controls (median and IQR; female: 14 and 8.75-21; male: 14 and 7–19). These findings indicate poorer dental status and reduced salivary secretion among psychiatric patients in comparison with healthy individuals.

When comparing the test and control groups, the psychiatric patients consistently showed higher xerostomia frequency and perceived caries susceptibility, together with lower UWS and labial MSG rates. Therefore, multiple linear regression analyses were performed to further investigate the potential effects of demographic variables and psychiatric medication use on salivary secretion and dental caries experience.

Table 6 presents the multiple linear regression analyses showing the effects of sex, age, and control/test group on salivary secretion and dental caries experience as assessed by the DMFT index. In case of UWS, sex showed positive association, indicating slightly higher values in male participants (weak correlation: partial-r = 0.141). For MSG labial secretion, age showed a negative association, suggesting reduced labial gland secretion with increasing age (weak correlation: partial-r = 0.224). The group variable was also significant, indicating lower labial MSG secretion among psychiatric participants (weak correlation: partial-r = 0.118). For MSG palatal secretion, the group effect was significant, with higher palatal MSG values observed in the psychiatric group (weak correlation: partial-r = 0.165). In case of DMFT, age emerged as a strong positive predictor indicating that it increased with age (strong correlation: partial-r = 0.651). The group variable also contributed significantly, with higher DMFT values in the psychiatric group (weak correlation: partial-r = 0.157).

**Table 6:** Multiple linear regression analyses for predictors of salivary secretion and dental caries experience

Dependent Variable	Factor	Unstandardized Coefficients B	95.0% Confidence Interval for B	Significance (*p*=)	Partial correlation
UWS (ml/min)	Sex	0.322	0.086–0.559	0.008**	0.141
	Age	0.006	0.000- 0.013	0.064	0.098
	Group	-0.146	-0.378- 0.087	0.219	-0.065
MSG labial (µl/cm^2^/min)	Sex	9.821	-9.746- 29.389	0.324	0.052
	Age	-1.226	-1.783-( -0.668)	0.000***	-0.224
	Group	-21.884	-41.133-( -2.636)	0.026*	-0.118
MSG palatal (µl/cm^2^/min)	Sex	0.309	-15.292- 15.910	0.969	0.002
	Age	-0.258	-0.703- 0.187	0.255	-0.060
	Group	24.539	9.205–39.872	0.002**	0.165
DMFT	Sex	-0.706	-1.931- 0.519	0.258	-0.060
	Age	0.287	0.252–0.322	0.000***	0.651
	Group	1.841	0.633–3.049	0.003**	0.157

The table presents the results of multiple linear regression models assessing the associations of demographic variables (sex, age) and psychiatric group status with unstimulated whole salivary secretion rate (UWS), minor salivary gland (MSG) secretion rates (labial and palatal), and dental caries experience (DMFT). Unstandardized regression coefficients (B), 95% confidence intervals, p-values, and partial correlation coefficients are shown. Partial correlation coefficients indicate the strength and direction of associations and were interpreted as weak (<0.3), moderate (0.3–0.6), or strong (≥0.6). Given the exploratory nature of the analyses, statistically significant results should be interpreted cautiously, particularly where effect sizes were small. Categorical predictors were entered as dummy variables (group: control = 1, psychiatric = 2; sex: female = 1, male = 2)

*Abbreviations*: *DMFT*Decayed, Missing and Filled Teeth index

Significant associations are indicated as bold and *p < 0.05, **p < 0.01, ***p < 0.001

Overall, these results highlight that age and psychiatric group were the most consistent predictors across the models, influencing both salivary secretion and DMFT.

As shown in Table 6, the multiple linear regression analyses identified significant differences within the psychiatric group. To better understand the underlying factors contributing to these effects, the psychiatric sample was further stratified by medication categories.

Table 7 summarizes the results of multiple linear regression analyses exploring the relationships between the different drug groups, demographic variables, XF and PCS. Analyses indicated that increased XF was associated with lower UWS values (weak correlation, partial-r = 0.109). The male sex (partial-r = 0.134), increasing age (partial-r = 0.132), and TA medication (partial-r = 0.162) were weekly associated with higher salivary flow rates. However, other medications had no significant effect on the UWS. TA was associated with decreased palatal MSG secretion (weak correlation, partial-r = 0.121), whereas taking BDZ (weak correlation, partial-r = 0.118) or LI (weak correlation, partial-r = 0.118) showed increased palatal MSG secretion. Labial MSG secretion showed a weak negative association with age, indicating a decline in secretion with increasing age (weak correlation, partial-r = 0.221). Analysis of the DMFT index revealed a strong positive correlation with age (partial-r = 0.651), suggesting that older individuals exhibited worse oral health. Furthermore, PCS (moderate correlation, partial-r = 0.307) SARI, (weak correlation, partial-r = 0.126) SNRI+NASSA (weak correlation, partial-r = 0.130) and LI (weak correlation, partial-r = 0.122) positively correlated with DMFT.

Overall, these analyses indicate that age was the most consistent predictor of oral health, while certain psychiatric medications exerted weak to moderate effects on salivary secretion parameters. Importantly, the subjective reports were consistent with the objective outcomes, as greater XF was associated with lower UWS, and higher PCS was accompanied by increased DMFT scores. 

**Table 7:** Predictors of salivary secretion and caries: regression analyses in psychiatric subgroups

Dependent Variable	Factor	Unstandardized Coefficients B	95.0% Confidence Interval for B	Significance (*p*=)	Partial correlation
UWS (ml/min)	Sex	0.303	0.064–0.543	0.013*	0.134
	Age	0.009	0.002–0.015	0.015*	0.132
	XF	-0.126	-0.249-(-0.004)	0.044*	-0.109
	PCS	-0.029	-0.135-0.078	0.59	-0.029
	SARI	-0.036	-0.628-0.556	0.905	-0.006
	SSRI	-0.174	-0.574-0.225	0.391	-0.046
	SNRI	-0.080	-0.578-0.418	0.752	-0.017
	NASSA	0.045	-0.570-0.660	0.886	0.008
	SNRI+NASSA	0.213	-0.907-1.333	0.708	0.020
	AA	-0.009	-0.314-0.295	0.952	-0.003
	TA	0.885	0.309–1.460	0.003**	0.162
	VPA	-0.180	-0.715-0.355	0.508	-0.036
	BD	-0.068	-0.394-0.257	0.679	-0.022
	LI	-0.049	-0.558-0.460	0.849	-0.010
	TCA	-0.218	-0.902-0.465	0.530	-0.034
MSG labial (µl/cm^2^/min)	Sex	10.214	-9.949-30.377	0.320	0.054
	Age	-1.236	-1.816-(-0.655)	0.000***	-0.221
	XF	-2.926	-13.275-7.424	0.57	-0.030
	PCS	-2.817	-11.809-6.175	0.538	-0.033
	SARI	-0.472	-50.468-49.291	0.985	-0.001
	SSRI	-10.352	-44.047-23.343	0.546	-0.033
	SNRI	13.236	-28.794-55.266	0.536	0.034
	NASSA	-2.626	-54.543-49.291	0.921	-0.005
	SNRI+NASSA	-5.849	-100.372-88.675	0.903	-0.007
	AA	-8.254	-33.936-17.429	0.528	-0.034
	TA	-43.164	-91.742-5.413	0.081	-0.094
	VPA	6.222	-38.930-51.374	0.787	0.015
	BDZ	-11.030	-38.477-51.374	0.430	-0.043
	LI	26.863	-16.105-69.830	0.220	0.066
	TCA	-31.667	-89.366-26.032	0.281	-0.058
MSG palatal (µl/cm^2^/min)	Sex	-2.147	-18.122-13.828	0.792	-0.014
	Age	-0.226	-0.686-0.234	0.334	-0.052
	XF	-6.132	-14.335-2.072	0.142	-0.079
	PCS	0.971	-6.160-8.101	0.789	0.014
	SARI	0.270	-39.384-39.925	0.989	0.001
	SSRI	-6.972	-33.694-19.750	0.608	-0.028
	SNRI	-5.902	-39.238-27.434	0.728	-0.019
	NASSA	-10.126	-51.304-31.051	0.629	-0.026
	SNRI+NASSA	-9.529	-84.500-65.443	0.803	-0.014
	AA	1.298	-19.070-21.667	0.900	0.007
	TA	-44.218	-82.746-(-5.689)	0.025*	-0.121
	VPA	28.316	-7.496-64.129	0.121	0.084
	BDZ	24.390	2.623–46.156	0.028*	0.118
	LI	38.028	3.948–72.107	0.029*	0.118
	TCA	-28.787	-74.550-16.977	0.217	-0.067
DMF-T	Sex	-0.611	-1.777-0.555	0.303	-0.056
	Age	0.270	0.237–0.304	0.000***	0.651
	XF	0.526	-0.078-1.130	0.088	0.092
	PCS	1.579	1.059–2.099	0.000***	0.307
	SARI	3.473	0.572–6.374	0.019*	0.126
	SSRI	0.684	-1.270-2.637	0.492	0.037
	SNRI	0.585	-1.850-3.020	0.637	0.026
	NASSA	1.298	-1.714-4.310	0.397	0.046
	SNRI+NASSA	-6.788	-12.278-(-1.299)	0.016*	-0.130
	AA	0.374	-1.117-1.865	0.622	0.027
	TA	-0.393	-3.195-2.409	0.783	-0.015
	VPA	-0.117	-2.673-2.439	0.928	-0.005
	BDZ	0.922	-0.669-2.513	0.255	0.061
	LI	-2.819	-5.265-(-0.373)	0.024*	-0.122
	TCA	-0.676	-4.027-2.674	0.692	-0.021

Multiple linear regression models were used to assess the associations of demographic variables (sex, age), subjective measures (xerostomia frequency [XF] and perceived caries susceptibility [PCS]), and specific psychiatric medication classes with unstimulated whole salivary secretion rate (UWS), minor salivary gland (MSG) secretion rates (labial and palatal), and DMFT within the psychiatric patient group. Unstandardized regression coefficients (B), 95% confidence intervals, p-values, and partial correlation coefficients are presented. Partial correlation coefficients reflect the strength and direction of associations and were interpreted as weak (< 0.3), moderate (0.3–0.6), or strong (≥ 0.6). Given the exploratory nature of these subgroup analyses and the generally small effect sizes observed, statistically significant findings should be interpreted cautiously and primarily as hypothesis-generating rather than evidence of drug-specific causal effects. Categorical variables were entered as dummy variables (sex: female = 1, male = 2; medication classes coded as present vs. absent)

*Abbreviations*: *SARI* Serotonin Antagonists and Reuptake Inhibitors, *SSRI* Selective Serotonin Reuptake Inhibitors, *SNRI* Serotonin–Norepinephrine Reuptake Inhibitors, *NASSA* Noradrenergic and Specific Serotonergic Antidepressants, *AA* Atypical Antipsychotics, *TA* Typical Antipsychotics, *VPA* Valproate, *BDZ*: Benzodiazepine, *LI* Lithium, *TCA* Tricyclic Antidepressants, *DMFT* Decayed, Missing, and Filled Teeth index

Significant associations are indicated as bold and *p < 0.05, **p < 0.01, ***p < 0.001

## Discussion

This study investigated subjective and objective indicators of oral dryness and salivation among psychiatric outpatients and healthy controls, with a particular focus on the salivary and dental consequences of the most frequently prescribed psychiatric medications. Our findings confirm that psychiatric patients experience increased xerostomia and poorer dental health, in line with previous research indicating that psychiatric conditions—together with their pharmacological treatment—elevate the risk of salivary disfunction and caries development [9–12]. Although patient selection was random, women were overrepresented in both the test (*n*** = **118, male *n* = 70) and control (*n* = 93, male *n* = 80) groups. This result is in harmony with previous reports indicating that women are more likely to seek psychiatric treatment and are typically overrepresented among psychiatric outpatients [43].

Previous research suggests that aging is not necessarily associated with reduced salivary gland output under normal conditions; however, decreased salivary flow is more commonly observed in older individuals in the presence of systemic diseases, medication use or increased functional demands on the salivary glands [11].

Interestingly, our findings only partially align with this trend: male patients demonstrated higher UWS rates compared with females, and increasing age weakly predicted higher UWS, whereas labial MSG secretion decreased with age. This contrasting pattern raises the possibility that major and minor salivary glands differ in their age-related susceptibility, or that compensatory mechanisms may help maintain baseline UWS despite declining minor gland secretion. These observations highlight the importance of assessing both major and minor salivary gland function when evaluating xerostomia in clinical populations.

At the same time, UWS was slightly reduced in psychiatric patients (females) compared with healthy controls. This finding corresponds with earlier reports indicating that chronic psychopharmacological treatment—particularly with drugs exerting anticholinergic or adrenergic effects—may suppress salivary output [15, 17, 29]. Although lower unstimulated whole saliva flow rates were anticipated primarily in psychiatric patients, relatively low UWS values were also observed in a subset of control participants. Under normal physiological conditions, unstimulated salivary flow rates typically range between 0.3 and 0.4 mL/min [44], whereas values below 0.1 mL/min are considered indicative of hyposalivation [45]. The unexpectedly low UWS values observed in controls may reflect several contributing factors. Despite predefined exclusion criteria, the presence of undiagnosed or unreported conditions affecting salivary secretion cannot be entirely excluded. In addition, previous studies have demonstrated that psychological stress may impair salivary gland function and reduce salivary flow [46, 47]. Accordingly, saliva collection in a dental care setting may have induced situational anxiety related to the anticipated dental examination, potentially leading to transient sympathetic activation and temporary changes in salivary composition and flow characteristics, as well as more difficult evacuation of saliva during collection using the spitting method. These observations suggest that reduced UWS values in controls may not necessarily indicate chronic salivary gland dysfunction and should therefore be interpreted with caution.

Within drug classes, typical antipsychotics showed a weak positive association with UWS in our cohort, which contrasts with classical descriptions of TA-related xerostomia [17]. This discrepancy may reflect heterogeneity in receptor profiles within the TA group or compensatory autonomic mechanisms emerging during long-term treatment.

Objective data on minor salivary gland secretion in psychiatric patients are scarce; therefore, our findings contribute to the existing literature with additional information: labial MSG secretion decreased with age and was lower in psychiatric patients, whereas palatal MSG secretion was higher in the psychiatric group, particularly among benzodiazepine and lithium users, while typical antipsychotics were associated with reduced palatal MSG secretion. These unexpected palatal findings may partly be explained by methodological challenges, as the palatal sampling site - typically near the upper first molar - can be difficult to identify precisely in the presence of missing molars, potentially introducing site-specific variability in secretion measurements [40]. At the same time, they may reflect differential drug effects across salivary gland types, given that minor salivary glands vary in receptor density and autonomic regulation. In addition, palatal minor salivary glands are known to exhibit a rapid, short-lasting secretory response followed by a refractory period, after which further secretion is temporarily reduced. This physiological characteristic may influence measured palatal flow rates depending on the timing of collection and local gland activity, and could therefore contribute to the relatively higher palatal secretion values observed in certain patient subgroups [42].

Beyond psychopharmacological mechanisms, salivary dysfunction has also been described in other medically complex populations characterized by chronic disease burden and autonomic dysregulation. Previous clinical studies in neurological and psychiatric disorders have demonstrated that xerostomia and altered salivary flow may coexist with compensatory glandular responses, and that subjective complaints often show only weak correlations with objective salivary measurements [48]. These observations are consistent with our findings and support a multifactorial clinical model in which salivary disturbances reflect not only medication effects but also disease-related autonomic alterations and adaptive mechanisms.

Psychiatric patients also reported significantly higher xerostomia frequency and perceived caries susceptibility. This pattern is consistent with earlier reports demonstrating that several psychiatric drug classes - particularly typical antipsychotics [17], serotonin antagonist and reuptake inhibitors [28], noradrenergic and specific serotonergic antidepressants [30], and serotonin–norepinephrine reuptake inhibitors [26] - are frequently associated with dry mouth.

In contrast, some authors have described SSRIs and SNRIs as less xerogenic due to their minimal affinity for peripheral muscarinic receptors [25, 27]. Our findings differ from these expectations, as subjective xerostomia was also commonly reported in these drug groups. This discrepancy supports the notion that xerostomia is not exclusively mediated by peripheral antimuscarinic effects, but may additionally involve central serotonergic mechanisms, drug combinations, cumulative receptor interactions, and subjective symptom perception.

Regarding dental status, psychiatric patients exhibited significantly higher DMFT scores than controls, consistent with earlier findings from Saudi Arabia [31], Serbia [32], and a related meta-analysis [1]. Age emerged as a strong predictor of DMFT in our cohort, in line with the cumulative nature of dental disease. Moreover, certain medication groups - including SARI, combined SNRI + NASSA therapy, and lithium - were associated with higher DMFT scores. This observation accords with previous evidence linking psychiatric medication use to increased caries risk through mechanisms such as hyposalivation, cognitive impairment, and behavioural changes [3, 49].

Only weak associations were observed between subjective xerostomia frequency and objective salivary flow parameters, however higher perceived caries susceptibility was accompanied by higher DMFT scores. This pattern suggests that subjective oral complaints might be accompanied by measurable clinical findings. Although several medication classes showed statistically significant associations with salivary and dental outcomes, the majority of these relationships were weak in magnitude. These findings support a multifactorial clinical model in which age, underlying psychiatric disease burden, and concomitant use of multiple psychiatric medications collectively contribute to alterations in salivary function and oral health status.

### Limitations

This study has several limitations that should be considered when interpreting the findings. First, no predefined minimum duration of psychiatric medication use was required for inclusion, and medication dosage was not incorporated into the analyses; therefore, potential dose–response relationships could not be assessed. Secondly, salivary secretion is influenced by multiple factors beyond psychopharmacological treatment, including stress, lifestyle factors, and general health status, which were not systematically controlled for in the present study. Health status in control participants was assessed by self-report questionnaire and interview on the day of saliva collection without additional medical screening, which may have limited the detection of unrecognized conditions influencing salivary flow. In addition, saliva sampling in the control group was performed in a dental care setting, often in close temporal proximity to the clinical examination, which may have induced situational stress and transiently affected unstimulated salivary flow rates. Due to the cross-sectional design, causal relationships between psychiatric medication use, salivary gland function, and oral health outcomes cannot be established.

## Conclusion

Psychiatric outpatients exhibited altered salivary secretion, more frequent xerostomia, and a higher caries burden compared with healthy controls. However, as unstimulated saliva collection in controls was conducted in a dental clinical setting, situational anxiety may have influenced flow rates, and future studies should consider separate control visits. Weak associations were observed between subjective complaints and objective measures, including lower UWS with higher xerostomia frequency and higher DMFT with greater perceived caries susceptibility. While age emerged as the strongest predictor of salivary and dental outcomes, the weak but notable associations observed for certain psychiatric drug groups reflect the multifactorial nature of oral health impairment in this population. Increased awareness of these risks among both psychiatric and dental professionals is essential for strengthening preventive strategies, early detection, and interdisciplinary care for individuals receiving long-term psychopharmacological treatment. Table 1. Overview of Psychiatric Medication Groups Used by Psychatric Patients. Table 2**.** Xerostomia and other associated subjective sicca symptoms questionnaire.

## Data Availability

The datasets generated and analysed during the current study are not publicly available due to their inclusion in ongoing research and future planned publications, but are available from the corresponding author on reasonable request.

## Abbreviations

AA: Atypical Antipsychotics
BDZ: Benzodiazepine
D: Dopamine receptor
DMFT: Decayed, Missing and Filled Teeth
GABA: Gamma-aminobutyric acid
H_1_: Histamine type 1 receptor
LI: Lithium
MAOI: Monoamine Oxidase Inhibitors
MSG: Minor salivary gland
n: Number of participants
NA: Noradrenalin
NASSA: Noradrenergic and specific serotonergic antidepressants
PCS: Perceived caries susceptibility
SARI: Serotonin Antagonist and Reuptake Inhibitors
SD: Standard deviation
SNRI: Serotonin-Norepinephrine Reuptake Inhibitor
SSRI: Selective Serotonin Reuptake Inhibitors
TA: Typical Antipsychotics
TCA: Tricyclic Antidepressants
UWS: unstimulated whole salivary secretion rate
VPA: Valproate
XF: Xerostomia frequency
5-HT: Serotonin
5-HT_2_: 5-hydroxytryptamine 2 receptor
α: Alfa adrenergic receptors
α_1_: Alfa_1_ adrenergic receptor
β: Beta adrenergic receptors
